# Demonstrative Midwifery—The Buffalo Medical Journal

**Published:** 1850-05

**Authors:** 


DEMONSTRATIVE MIDWIFERY—THE BUFFALO MEDICAL JOURNAL.

Our valued and esteemed cotemporary, of the Buffalo Medical Journal, seems to have roused quite a professional warfare, by his approval of a specimen of demonstrative midwifery, exhibited before the graduating class of the medical department of the University of Buffalo. And we notice, too, in connection with this controversy, that the clergy, with their usual instincts and propensities for medical matters, have stepped forward to take a part in the affair, so that our friend Flint, if he should die in the warfare, will have the gloomy consolation of dying with the benefit of clergy.

We are really at a loss to understand the reasons for the extraordina ry position assumed by the seventeen physicians whose names are appended to a letter, which appeared in the March number of the Buffalo Medical Journal, in which letter there is a bitterness of denunciation altogether unwarranted we think, by the circumstances. Such strong terms as "unprofessional in manner, and grossly offensive, alike to morality and common decency," are rather strong for use towards gentlemen, quite as able as the letter writers to appreciate what is professional, and what accords with morality and common decency. In our judgment, the letter writers agonized the matter quite enough, to say the least.

That there are circumstances which would make demonstrative midwifery open to such denunciations as we have quoted, we readily admit, but one of the plainest and most sensible rules of logic forbids us from arguing against the use of anything, from its abuse. As far as "we understand" the case that drew forth the denunciatory letter, there was no departure whatever from professional manner, nor from "morality or common decency." These are relative terms; what might be consid ered very indecent by one virtuous female, by another, might be looked upon in a different light. In the instance before us, the female acted voluntarily; no improper means were taken to procure her consent, and she was satisfied and pleased with the professional ministrations she received. Surely her judgment is worth something in a matter of this kind; may we say that her satisfaction far outweighs the ponderous displeasure of her volunteer champions? There is nothing in the curriculum of a college that needs demonstrative teaching more than midwifery, and we regret that the opportunities for demonstration are so very limited. The seventeen physicians of Buffalo are evident ly not disposed to enlarge its means, and their views savor very much of the popular prejudices against dissections.

Dr. Bennet speaks very sensibly on a kindred subject, and his remarks may well be applied to the matter before us. He says: "In Paris hospital practice, the objections which exist in England to examination by the touch, or by the speculum, either are not met with, or are not allowed by those physicians and surgeons who pay special attention to uterine diseases; consequently, little more difficulty is experienced in appreciating, by their means, the symptoms furnished by the uterine organs, than in resorting to any usual means of investigation in diseases of other parts of the economy."

The indisposition of English physicians to investigate uterine disorders is thus referred to by Dr. Bennet: "That this laudable sense of propriety is, however, often carried much too far by the members of the medical profession with us, is well known to all who specially study uterine pathology." Is the practice thus commented on calculated to advance or retard medical science, or its healing powers? The answer is obvious, and that answer meets the case in the medical college of Buffalo.

We cannot withhold from Dr. Austin Flint the expression of our warm approval of his dignified, professional course. That he has the right side of the questiou, the strength of argument, and the force of professional precedents, we are well convinced.

